# Insights into genetic determinants of piglet survival during a PRRSV outbreak

**DOI:** 10.1186/s13567-024-01421-8

**Published:** 2024-12-18

**Authors:** Joaquim Tarrés, Teodor Jové-Juncà, Carles Hernández-Banqué, Olga González-Rodríguez, Llilianne Ganges, Sofia Gol, Marta Díaz, Josep Reixach, Ramona N. Pena, Raquel Quintanilla, Maria Ballester

**Affiliations:** 1 Animal Breeding and Genetics Program, Institute of Agrifood Research and Technology (IRTA), Caldes de Montbui, Spain; 2 Centre de Recerca en Sanitat Animal (CReSA), Unitat Mixta d’Investigació IRTA-UAB en Sanitat Animal, Campus Universitat Autònoma de Barcelona (UAB), 08193 Barcelona, Bellaterra Spain; 3 Selección Batalle SA, Riudarenes, Spain; 4 Departament de Ciència Animal, University of Lleida and AGROTECNIO-CERCA Center, Av. Rovira Roure 191, 25198 Lleida, Spain

**Keywords:** PRRSV, disease, survival, immunity traits, genetic markers, SNP, immune response

## Abstract

Breeding animals to produce more robust and disease-resistant pig populations becomes a complementary strategy to the more conventional methods of biosecurity and vaccination. The objective of this study was to explore the ability of a panel of genetic markers and immunity parameters to predict the survival rates during a natural PRRSV outbreak. Ten-week-old female Duroc pigs (*n* = 129), obtained from 61 sows and 20 boars, were naturally infected with a highly pathogenic PRRSV genotype 1 strain. Prior to infection, piglets were screened for immunity parameters (IgG levels in plasma and *SOX13* mRNA expression in blood) and genetic markers previously associated to PRRSV immune response and immunity traits. Additionally, the 20 boars were genotyped with a panel of 132 single nucleotide polymorphisms (SNPs). Survival analysis showed that mortality was significantly higher for animals with low basal IgG levels in plasma and/or high *SOX13* mRNA expression in blood. The genotypes of sires for SNPs associated with IgG plasma levels, CRP in serum, percentage of γδ T cells, lymphocyte phagocytic capacity, total number of lymphocytes and leukocytes, and MCV and MCH were significantly associated with the number of surviving offspring. Furthermore, *CD163* and *GBP5* markers were also associated to piglet survival. The effects of these SNPs were polygenic and cumulative, survival decreased from 94 to 21% as more susceptible alleles were accumulated for the different markers. Our results confirmed the existence of genetic variability in survival after PRRSV infection and provided a set of genetic markers and immunity traits associated with PRRS resistance.

**Supplementary Information:**

The online version contains supplementary material available at 10.1186/s13567-024-01421-8.

## Introduction

Infectious diseases are a major threat to the sustainability and profitability of livestock production, global food security and public health. Additionally, they contribute to the growing challenge of antimicrobial resistance. The intensification of the swine industry, coupled with the ever-increasing movement of pigs and pork products worldwide, facilitates the emergence and spread of infections pathogens. In this scenario, breeding animals to produce more robust and disease-resistant pig populations becomes a complementary strategy to the more conventional methods of biosecurity, vaccination, and treatment [1].

One of the major infectious challenges worldwide for pigs is porcine respiratory and reproductive syndrome (PRRS), caused by the porcine reproductive and respiratory syndrome virus (PRRSV). The PRRSV is a small, enveloped, single-stranded positive-sense RNA virus divided into two genotypes: PRRSV-1 (species Betaarterivirus suid 1 or EU) and PRRSV-2 (species Betaarterivirus suid 2 or US) with only 50–60% nucleotide identity [2]. In addition to genotype differences, varying host immune responses have been described depending on the PRRSV pathogenicity, as well as other factors including the age, nutritional and health status of pigs [3–5]. Furthermore, several studies have demonstrated the existence of genetic variability in susceptibility/resistance of pigs against PRRSV infection, with the identification of QTLs, genes and genetic variants associated with different PRRS phenotypes [5–10].

Currently, there are more than 100 QTLs with associated genetic markers for PRRS viral load, PRRSV antibody titer, and PRRSV susceptibility described in the PigQTLdb [11]. The first molecular marker identified within a major QTL on Sus scrofa chromosome 4 (SSC4) for PRRSV resistance and productivity was the WUR10000125 (rs80800372) polymorphism located in the 3’UTR region of the *GBP1* gene [8]. Later, the causality of this QTL was attributed to a nearby gene, *GBP5*, where an intronic SNP (rs340943904) introduces a splice acceptor site, changing the proportions of *GBP5* transcripts levels [12]. The GBP family are interferon-induced GTPases which play a role in protective immunity against pathogens through cell-autonomous defense and inflammasome-driven responses [13]. Natural mutations in the *CD163* gene have also been associated to changes in the susceptibility of pigs to PRRSV infection and enhanced weight gain [10, 14–17]. *CD163* encodes a member of the scavenger receptor cysteine-rich (SRCR) superfamily and is exclusively expressed in cells from the monocyte/macrophage lineage. CD163 has been recognized as an essential receptor used by the PRRSV for entry into macrophages and initiate infection [18]. Deletion by genetic editing of this receptor makes pigs resistant to PRRSV infection [19, 20]. Genetic polymorphisms in other genes related to host immune responses such as *MX1* or *SGK1* have also been associated to susceptibility/resistance to PRRSV infection [7].

Therefore, selecting PRRSV-resistant pigs is feasible, considering the existence of natural genetic variation. Additionally, the inclusion of genetic markers in breeding programs to enhance the overall immunocompetence of animals will facilitate the selection of animals for disease resilience against a wide variety of pathogens [21, 22]. The genetic determinism of innate and adaptive immunity traits has previously been determined, describing medium to high heritabilities for most of the analysed traits [23–26]. Furthermore, in a previous study performed by our group, wherein a set of 30 related traits covering immune, haematological, and stress parameters were measured in healthy Duroc pigs at 60 ± 8 days of age, we identified 40 significantly associated SNPs at whole-genome level for IgG, γδ T-cells, C-reactive protein, lymphocytes phagocytic capacity, total number of lymphocytes, leukocytes and neutrophils, mean corpuscular volume and mean corpuscular haemoglobin [25].

In this study, we have explored the ability of a panel of genetic markers, including previously reported PRRSV immune response markers and markers associated to innate and adaptive traits, to predict the survival rates during a natural PRRSV outbreak. Furthermore, the relationship between these genetic markers and some carcass traits obtained at the slaughterhouse was also evaluated.

## Materials and methods

### Animal material

A total of 129 female pigs from a commercial Duroc pig line were used for this study. The pigs stayed in six consecutive pens (21 ± 1 animals per pen) and belonged to 61 litters (one to three piglets per litter) obtained from 61 sows and 20 boars. Piglets were well distributed across pens, as half of the sires had daughters placed in 5–6 different pens, ensuring good genetic connection between pens. All animals came from a negative sow farm and were raised in the same farm and fed ad libitum with a commercial cereal-based diet. All pigs were apparently healthy, without any sign of infection when samples of blood were collected at 60 ± 2 days of age from all animals. Blood was collected via the external jugular vein into vacutainer tubes with anti-coagulants (Sangüesa S.A., Spain), according to the requirements for further immunity measurements, and Tempus^™^ Blood RNA tubes (Thermo Fisher Scientific, Spain) to stabilize the RNA. All samples were transported with ice blocks to the laboratory and stored at −20 °C or −80 °C for further processing.

One week later, the ten-week-old female Duroc pigs were naturally infected with a highly pathogenic PRRSV genotype 1 strain. On the first day of infection, we observed some sick animals, and the next day, lung swabs were collected from two dead animals for sequencing of the *ORF5* gene of the PRRSV, which tested positive. This virus strain (Rosalia) is highly virulent, and due to the small size of the farm, the infection spread rapidly, with all pigs becoming infected within 1–2 days. The infection lasted for 6 weeks and resulted in a mortality rate of 47 dead pigs, with 82 animals surviving at 15 weeks (when the animals were moved to the fattening farm). Blood samples from 113 of the 129 animals were collected one week after the first symptoms appear and viral RNA was isolated from 88 samples using the MagAttract 96 *cador* Pathogen kit (Qiagen, Spain). PRRSV infection was confirmed through RT-qPCR laboratory analyses using the VetMAXTM PRRSV EU & NA 2.0 kit (Thermo Fisher Scientific, Spain).

The 82 surviving animals were fattened in the same farm with identical feeding and management conditions. The animals were fed ad libitum with a commercial cereal-based diet and were apparently healthy despite four more animals died during this period. The remaining 78 animals were slaughtered at an average weight of 137 kg, ageing between 245 and 262 days, and belonged to 43 litters obtained from 18 boars and 43 sows. All animals from 31 out of 43 litters survived the PRRS outbreak. All animals from the other litters, up to 61, died during the outbreak, and therefore, these 18 litters did not have measurements at slaughter.

After slaughter, the hot carcass weight was measured. Carcass lean meat percentage and lean meat percentage of the main retails of the carcass (ham, loin and shoulder) were estimated using an online ultrasound automatic scanner (AutoFOM, Frontmatec Group, Kolding, Denmark). The carcass lean percentage was estimated based on measurements of 16 ultrasonic transducers that scanned the carcass every 5 mm. The same equipment also provided estimations of backfat thickness and loin thickness at 6 cm off the midline between the third and fourth last ribs, as well as backfat thickness in the ham. The measurements of animals arriving at the slaughterhouse were averaged per litter. For a few litters, data from daughters not included in the study were also considered.

**Figure 1:**
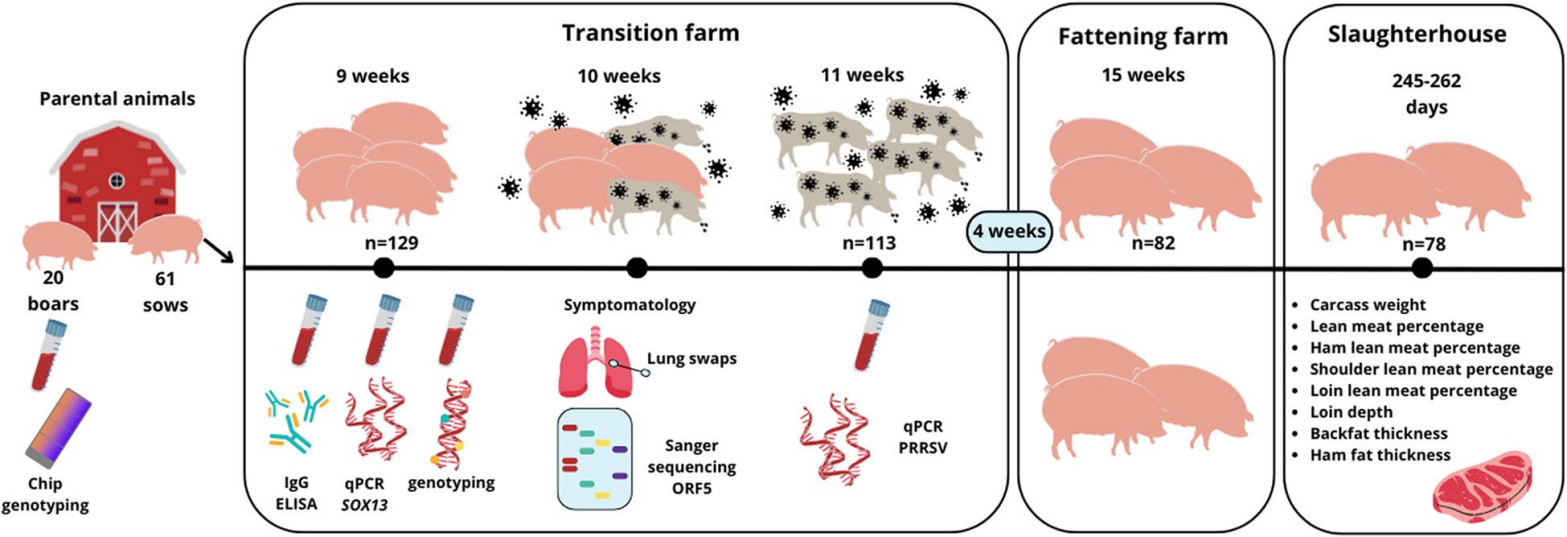
**Chronogram of the study. Created using Canva software**. All assets used were sourced from Canva’s license-free library.

Figure 1 shows the timeline of the study, including the number of animals used and samples collected.

### Phenotypic and genetic analyses

The 129 pigs were screened for immunity parameters and biomarkers. Total concentration of immunoglobulins IgG in plasma was measured by ELISA with commercial kits (Bethyl laboratories Inc., Bionova, Spain), following the manufacturer’s instructions. Plasma was collected from blood sampled in 6 mL heparinised tubes and diluted 1:50 000 to detect IgG. Samples, in duplicate, were quantified by interpolating their absorbance from the standard curves constructed with known amounts of pig IgG and corrected for sample dilution. Absorbance was read at 450 nm using a microplate reader (LUMistar Omega, BMG Labtech, Germany) and analysed using the Omega MARS software (BMG Labtech, Germany).

The mRNA expression levels of *SOX13* gene, an essential γδ T-cell transcription factor, were quantified by qPCR. Whole blood RNA was isolated from Tempus tubes using Tempus^™^ Spin RNA Isolation Reagent Kit (Thermo Fisher Scientific, Spain) and quantified using Nanodrop ND-1000 spectrophotometer. One μg of RNA was reverse-transcribed into cDNA using the PrimeScript RT Reagent Kit (Takara, Condalab, Spain) in a total volume of 20 μL, following the manufacturer’s instructions. *SOX13* primer pair (F-5′-AAGCCAAAGACGTCAAAGGGA-3′ and R-5′-TCCCGAAGGGTGGACAGTT-3′) was designed using PrimerExpress 2.0 software (Applied Biosystems). The *β2M* and *HPRT1* genes were used as endogenous controls [27]. A QuantStudio^™^ 12 K Flex Real-Time PCR System (Thermo Fisher Scientific) was used for mRNA quantification using SYBR Green chemistry (SYBR™ Select Master Mix, Thermo Fisher Scientific, Spain). The reactions were carried out in a 384-well plate in 15 μL volume containing 3.75 μL of cDNA sample diluted 1/20. Primer concentration was 300 nM. The thermal cycle was: 10 min at 95 °C, 40 cycles of 15 s at 95 °C and 1 min at 60 °C. Each sample was analysed in duplicate. Data was analysed using the Thermo Fisher Cloud software (Applied Biosystems) and the comparative Ct method [28]. The sample with the lowest *SOX13* expression was selected as calibrator.

Genomic DNA was isolated from EDTA-collected blood samples using a chemagic 360 instrument with DNA blood 250 Kit H96 (PerkinElmer, Baesweiler, Germany). DNA concentration and purity were measured in a Nanodrop ND-1000 spectrophotometer. The 129 pigs were screened for the following genetic markers using qPCR-HRM (high-resolution melting): *GBP5* (rs340943904), *CD163* (rs1107556229), *SGK1* (rs338508371), and *MMRN1* (rs695254451) [7, 10]; allelic discrimination using allele-specific Taqman probes: *CRP* (rs341595340 and rs327446000) [29], and end-point PCR protocol: *MX1*_c.−547ins + 275 [10].

### Sire DNA extraction and SNP genotyping

Genomic DNA was extracted from blood samples using the NucleoSpin Blood kit (Macherey–Nagel, Düren, Germany). DNA concentration and purity were measured in a Nanodrop ND-1000 spectrophotometer.

The 20 boars were genotyped with a custom commercial panel of 128 SNPs using a custom-designed *TaqMan* OpenArray genotyping plate in a QuantStudio^™^ 12 K flex Real-Time PCR System (Thermo Fisher Scientific). This SNP chip, used for paternity control, was modified to include some tag SNPs associated with several health and productivity traits. Additionally, the boars were genotyped for the following genetic markers: *GBP5* (rs340943904), *CD163* (rs1107556229), *MX1*_c. −547ins + 275, *SGK1* (rs338508371) and *MMRN1* (rs695254451) as previously described.

### Statistical analysis

An exploratory analysis of the phenotypes (IgG and *SOX13*) was carried out for investigating both the raw data distribution and the best fitting model for subsequent analyses. Systematic non-genetic putative effects (censoring, pen, and sire) on IgG and *SOX13* traits were tested by using a linear model. Statistical analyses were performed using R software.

### Piglet survival analysis

The survival time *t* of a piglet was calculated as the difference in days between the date of birth and the date of death. The records of piglet still alive at the end of the study were regarded as censored (at 15 weeks of age, i.e. 105 days). After editing, our database included data on 47 dead pigs (36.4% mortality) and 82 surviving animals (63.6% survival). Kaplan–Meier survival functions [30] were estimated stratifying by pen, IgG and *SOX13* levels.

### Animal markers association studies with piglet survival

The risk of dying was analysed under the following semi-parametric proportional hazards model:

$$h\left({t}_{ij}\right)={h}_{0}({t}_{ij})exp\left({pen}_{j}+{l}_{l}+{u}_{i}+{s}_{ik} {a}_{k}\right)$$

where h(t) was the hazard function at time t and ${h}_{0}(\text{t})$ was the unknown baseline hazard function. In the exponential term, all effects were assumed to be time-independent (or proportional); ${pen}_{j}$ corresponded to the jth pen effect (6 levels); ${l}_{l}$ was the litter effect of sow I, with $l\sim N(0,{\sigma }_{l}^{2})$ and ${\sigma }_{l}^{2}$ was the litter variance; ${u}_{i}$ was the infinitesimal genetic effect of animal i, with $u\sim N(0,A{\sigma }_{u}^{2})$, where $\text{A}$ is the numerator relationship matrix computed on the basis of pedigree (1388 individuals, five generations) and ${\sigma }_{u}^{2}$ is the additive genetic variance; ${\text{s}}_{\text{ik}}$ is the animal genotype (coded as 0,1,2) for the kth SNP, and ${\text{a}}_{\text{k}}$ was the allele substitution effect of the SNP on the risk of dying. The genotyped SNPs were included in the model one at a time. Estimation of the model variance components for piglet survival and the corresponding heritability ${h}^{2}={\sigma }_{u}^{2}/\left[{{\sigma }_{u}^{2}+\sigma }_{l}^{2}+1\right]$ was performed using the Survival kit program R package [31].

The piglet survival function for each SNP genotype ${\text{s}}_{\text{ik}}$ can be calculated as

$$S\left(t\right)={{S}_{o}(t)}^{{HR}_{k}}$$

where the hazard ratio is ${HR}_{k}=\text{exp}({a}_{k})$ and ${S}_{0}\left(t\right)$ is the reference survival function. The SNPs included in the model were: *GBP5* (rs340943904), *CD163* (rs1107556229), *MX1*_c.−547ins + 250, *MMRN1* (rs695254451) and *CRP* (rs341595340 and rs327446000).

### Sire markers association studies with daughters’ survival

Association analysis was carried out between the risk of dying and the 132 SNPs genotyped in the boars. For this purpose, the Survival kit program R package was employed to fit the following semi-parametric proportional hazards model:

$$h\left({t}_{ij}\right)={h}_{0}({t}_{ij})exp\left({pen}_{j}+{l}_{l}+{u}_{i}+{s}_{ik} \;{a}_{k}\right)$$

where h(t) was the hazard function at time t and ${h}_{0}(\text{t})$ was the unknown baseline hazard function. In the exponential term, all effects were assumed to be time-independent (or proportional); ${pen}_{j}$ corresponded to the jth pen effect (6 levels)); ${l}_{l}$ was the litter effect of sow I, with $l\sim N(0,{\sigma }_{l}^{2})$ and ${\sigma }_{l}^{2}$ was the litter variance; ${u}_{i}$ was the infinitesimal genetic effect of animal i, with $u\sim N(0,A{\sigma }_{u}^{2})$, where $\text{A}$ is the numerator relationship matrix computed on the basis of pedigree (1388 individuals, five generations) and ${\sigma }_{u}^{2}$ is the additive genetic variance; ${\text{s}}_{\text{ik}}$ is the sire genotype (coded as 0,1,2) for the kth SNP, and ${\text{a}}_{\text{k}}$ was the allele substitution effect of the SNP on the risk of dying. The SNPs were included in the model one at a time.

### Cumulative effect of genetic markers on survival to PRRS

The eleven markers significantly associated with immunity and survival to PRRS were selected to generate a global immunocompetence index. A value of 0 was assigned to the allele resistant to PRRS, while 1 was assigned to the allele susceptible to PRRS. For each sire, a global immunocompetence index value was obtained by summing the alleles multiplied by the substitution effect of each marker.

$${index}_{i}=\sum_{k}{s}_{ik} {a}_{k}$$

The substitution effects were estimated by fitting the following proportional hazards model:

$$h\left({t}_{ij}\right)={h}_{0}({t}_{ij})exp\left({pen}_{j}+{l}_{l}+{u}_{i}+\sum_{k}{s}_{ik}\; {a}_{k}\right)$$

where h(t) was the hazard function at time t and ${h}_{0}(\text{t})$ was the unknown baseline hazard function. In the exponential term, all effects were assumed to be proportional; ${pen}_{j}$ corresponded to the jth pen effect (6 levels); ${l}_{l}$ was the litter effect of sow I, with $l\sim N(0,{\sigma }_{l}^{2})$; ${u}_{i}$ was the infinitesimal genetic effect of animal i, with $u\sim N(0,A{\sigma }_{u}^{2})$, where $\text{A}$ is the numerator relationship matrix computed on the basis of pedigree (1388 individuals, five generations) and ${\sigma }_{u}^{2}$ is the additive genetic variance; ${\text{s}}_{\text{ik}}$ is the sire genotype (coded as 0,1,2) for the kth SNP, and ${\text{a}}_{\text{k}}$ was the allele substitution effect of the SNP on the risk of dying.

### Association of global immunocompetence index with slaughter measurements post-infection

This global immunocompetence index was fitted as an effect in univariant mixed animal models for each slaughter measurements post-infection as follows:

$${y}_{l}=\mu +{u}_{l}+{b*index}_{l}+{e}_{l}$$

where ${y}_{l}$ were slaughter measurements post-infection for each litter; $\upmu$ was the intercept of these phenotypes; ${u}_{l}$ was the infinitesimal genetic effect of litter l, with $u\sim N(0,A{\sigma }_{u}^{2})$, where $\text{A}$ is the numerator relationship matrix computed on the basis of pedigree and ${\sigma }_{u}^{2}$ is the additive genetic variance; ${index}_{l}$ is the global immunocompetence index of the sire and *b* is its regression coefficient effect; ${e}_{l}$ was the random error effect of litter l, with $e\sim N(0,{\sigma }_{e}^{2}/{\upomega }_{l})$, where ${\sigma }_{e}^{2}$ is the residual variance and ${\upomega }_{l}$ an associated weight indicating the amount of information summarized for that litter (i.e. the number of daughters arriving at slaughterhouse). Genetic parameters were estimated by restricted maximum likelihood using the BLUPF90 software package [32].

## Results

### Detection and characterization of PRRSV in the animals during the study

Ten-week-old female Duroc pigs were naturally infected with the highly pathogenic PRRSV-1 Rosalia strain, as confirmed by sequencing the *ORF5* gene of PRRSV (Additional file 1). One week later, viral levels of 88 individuals were analysed from whole-blood samples by qPCR, indicating that all animals were positive and 88% of pigs had high amounts of the virus (Ct < 25). The mortality of this outbreak after 6 weeks reached 36.4% (47 dead from 129 pigs). No association was found between PRRSV viral load and mortality.

### Descriptive statistics

Plasma IgG levels and *SOX13* mRNA expression levels in blood were quantified before the outbreak took place in apparently healthy animals showing no signs of infection. Table 1 shows descriptive statistics for IgG levels and *SOX13* gene expression in blood in our studied Duroc population. Dead pigs had significantly higher levels of *SOX13* gene expression than the surviving animals. The infection originated in the third pen, where the IgG levels where significantly lower compared to other pens. This pen experienced over 50% of piglet mortality. Similarly, the sixth pen also had around 50% mortality. Notably, in the sixth pen, pigs had significantly higher levels of *SOX13* gene expression. There were huge differences in piglet survival depending on its sire. Out of the 20 sires, eight had almost not daughter mortality, with only 2 deaths out of 44 piglets. Conversely, five sires had over 80% of mortality, with 23 deaths out of 28 piglets. Remarkably, these 28 piglets had lower levels of IgG and significantly higher levels of *SOX13* gene expression (Table 1).

**Table 1:** **Descriptive statistics of the analysed immunity parameters and piglet survival traits**

Variable	n	IgG^1^	SOX13^1^	Deaths	Survival
Censoring					
Alive	82	4.84^a^	6.42^a^	0	1.00^a^
Deads	47	4.44^a^	7.80^b^	47	0.00^b^
Pen					
1	22	5.09^a^	6.92^ab^	5	0.77^a^
2	21	4.68^ab^	5.99^a^	9	0.57^ab^
3	20	3.74^b^	6.84^ab^	11	0.45^b^
4	22	4.35^ab^	6.48^a^	7	0.68^ab^
5	22	5.56^a^	6.41^a^	4	0.82^a^
6	22	4.68^ab^	8.85^b^	11	0.50^b^
Sire					
Low survival (*n* = 5)	28	3.97^b^	9.73^b^	23	0.18^b^
Medium survival (*n* = 7)	57	4.66^a^	6.51^a^	22	0.61^ab^
High survival (*n* = 8)	44	4.73^a^	6.82^a^	2	0.95^a^

^1^Only 128 and 122 animals have values of IgG and *SOX13* gene expression in blood, respectively

^a,b^Estimates with different letter superscripts within a column are significantly different at a nominal *P* < 0.05

### Piglet survival analysis

**Figure 2:**
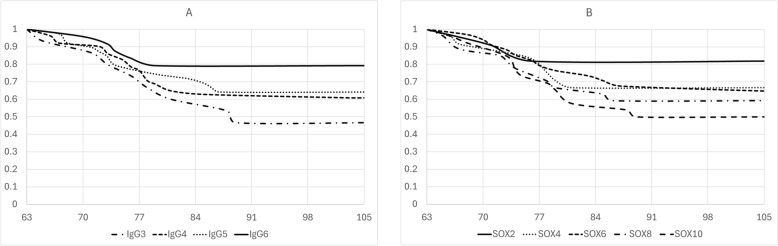
**Kaplan–Meier survival functions stratified by each immunity trait.** **A** IgG levels were categorized as: (IgG3 = IgG < 3.0, IgG4 = 3.0 < IgG < 4.5, IgG5 = 4.5 < IgG < 6.0, IgG6 = IgG > 6.0). **B** SOX13 mRNA expression levels were categorized as: (SOX2 = SOX13 < 3.0, SOX4 = 3.0 < SOX13 < 5.0, SOX6 = 5.0 < SOX13 < 7.0, SOX8 = 7.0 < SOX13 < 9.0, SOX10 = SOX13 > 9.0).

Kaplan–Meier survival functions show the effects of IgG and *SOX13* gene expression levels on piglet survival (Figure 2). Animals with IgG levels higher than 6 had 3.25 times lower risk of dying than animals with IgG levels below 3. This difference was significant, resulting over 30% higher mortality by the end of the study (79.2% vs 46.7% piglet survival) (Figure 2A). Animals with *SOX13* gene expression levels over 9 had 3 times higher risk of dying than animals with *SOX13* levels under 3. This difference was also significant, resulting in over 30% higher mortality at the end of the study (81.8% vs 50.0% piglet survival) (Figure 2B).

### Genetic parameters of piglet survival

The genetic determinism of piglet survival to PRRS was first explored by the heritability of this trait. Given an estimated genetic variance of 1.125 and a litter variance of 0.874, the heritability estimate of piglet survival to this PRRS outbreak was 0.375. Despite typically survival has low heritability, our results show higher levels of heritability because of the high quality of the dataset where all animals were infected, the mortality was very high and it was due to an unique cause [33].

### Genetic markers associated to piglet survival

The genotypes of the animals for the *CD163* and *GBP5* markers were significantly associated with the number of surviving offspring (Table 2). In both markers, the GG genotypes had over two times more risk of dying than genotypes AA and GT, respectively. Animals AA for *CD163* had significantly higher survival up to 15 weeks than animals GG (74% vs 42%). Animals with the GT genotype for *GBP5* also had significantly higher survival than animals with the GG genotype (70% vs 58%). The interaction between both markers was even more significant (Table 3); animals with genotype AA for *CD163* and GT for *GBP5* had over 90% survival rate. Conversely, animals with the GG genotype for both markers had a 14 times higher risk of mortality than those with other genotypes, and only 35% of animals in this category survived during the outbreak. The polymorphisms of *CRP*, *MMRN1*, and *MX1* genes were not associated with the number of surviving animals. The *SGK1* genetic marker rs338508371 was not segregating in the Duroc population. The allele and genotype frequencies of these genetic markers were in Hardy–Weinberg equilibrium.

**Table 2:** **Description of the genetic markers associated with survival up to 105 days after the PRRS outbreak depending on piglet genotype**

Assay name	Allele (frequency)				Genotype (survival up to 105 days)^3^		
	Gene	Resistant	Susceptible	LRT^1^	Homozygous resistant	Heterozygous	Homozygous susceptible
rs1107556229	*CD163*	A (0.58)	G (0.42)	5.88 *	AA (0.74)	AG (0.68)	GG (0.42)
rs340943904	*GBP5*	T (0.20)	G (0.80)	4.01 *	TT (1.00)	GT (0.70)	GG (0.58)
MX1_c.−547ins + 250	*MX1*	D (0.78)	I (0.22)	2.02 ns	DD (0.68)	ID (0.55)	II (0.59)
rs695254451	*MMRN1*	T (0.29)	C (0.71)	2.57 ns	TT (0.72)	TC (0.67)	CC (0.58)
rs341595340^2^	*CRP*	A (0.93)	C (0.07)	0.66 ns	AA (0.68)	AC (0.56)	

^1^*P*-value for the likelihood ratio test of proportional hazard models including or not each SNP: *** = *P* < 0.001, ** = 0.001 < *P* < 0.01, * = 0.01 < *P* < 0.05, +  = 0.05 < *P* < 0.10, ns = *P* > 0.10

^2^*CRP* polymorphisms (rs341595340 and rs327446000) were found to be in complete LD with each other

^3^Predicted proportion of animal still alive at 105 days depending on their genotype for each SNP: animals with two resistant alleles (Homozygous resistant), animals with one resistant and one susceptible allele (Heterozygous), and animals with two susceptible alleles (Homozygous susceptible)

**Table 3:** **Hazard ratios and survival up to 105 days depending on the interactions of genotypes for *CD163* and** ***GBP5*** **genes**

Genotypes		Animals	Deaths	Hazard ratio	Survival up to 105 days
*GBP5*	*CD163*				
TT	AG	2	0	0.00	1.00^a^
GT	AA	14	1	1.00	0.93^a^
GT	AG	26	9	4.57	0.72^ab^
GT	GG	7	3	6.54	0.62^ab^
GG	AA	24	8	5.35	0.68^ab^
GG	AG	44	19	7.04	0.60^b^
GG	GG	12	7	14.39	0.35^c^

^a,b,c^Estimates with different letter superscripts indicate that survival up to 105 days are significantly different at a nominal *P* < 0.05

### Sire markers associated with survival of daughters to PRRS

To assess the effect of other genetic markers associated to health-related traits [25] on piglet survival to PRRS, an association study was performed using the mortality risk of the 129 Duroc pigs and the genotypes of 132 SNPs of their 20 sires. The full list of associated SNPs, with their predicted consequences on daughters’ survival is shown in Table 4. It is worth to highlight that 10 out of 12 SNPs described by [25] and related to immunity traits were significantly associated with daughters’ survival to PRRS (Table 4). These SNPs were in five chromosomal regions on pig chromosomes SSC4, SSC6, SSC12, SSC17 and SSCX. The rs319560097 SNP was associated with IgG plasma levels. Boars with the TT genotype had daughters with higher IgG plasma levels and had significantly higher survival up to 15 weeks than daughters of boars with the CT genotype (100% vs 58%). The SNP rs81233340 was associated with CRP levels. Boars TT had significantly higher daughters’ survival up to 15 weeks than boars TG (69% vs 33%). Two SNPs, rs338661853 and rs80904079, located in two regions of SSC6 and associated with lymphocytes phagocytic capacity and mean corpuscular volume (MCV) and mean corpuscular haemoglobin (MCH) traits, respectively, were also identified as associated with daughters’ survival to PRRS. Boars with the GG genotype for rs338661853 had significantly higher daughters’ survival up to 15 weeks than boars with the AG genotype (74% vs 36%). Regarding the rs80904079 SNP, boars with the AA genotype had significantly higher daughters’ survival up to 15 weeks than boars with the AG genotype (71% vs 45%). Three of the boars with the GG genotype were also TT for the rs319560097 SNP, and all of their daughters survived. The rest of boars with the GG genotype for the rs80904079 SNP had lower survival than boars with the AA genotype. In terms of leukocytes count in blood, the associated SNP rs323856019 at 3.24 Mb on SSC12 was also associated with survival to PRRS. Boars with the CC genotype had significantly higher daughters’ survival up to 15 weeks than boars with the TC genotype (75% vs 55%). In SSC17, three SNPs (rs80803525, rs80924885, rs80899023) located within the genomic interval at 52.46–52.51 Mb were associated with lymphocytes count in blood and PRRS survival. These SNPs were in linkage disequilibrium. Boars TC for SNP rs80803525 had significantly higher daughters’ survival up to 15 weeks than boars CC (79% vs 58%). Half of the boars with the TT genotype for SNP rs80803525 were also GG for SNP rs338661853, which is associated with lymphocytes phagocytic capacity, and their daughters’ survival up to 15 weeks was 75%. The remaining boars with the TT genotype had only a 40% of daughters’ survival. These boars all had the AG genotype for SNP rs338661853. The percentage of γδ T cells-associated SNP rs342772739, located within 33.51–33.64 Mb of SSCX, was found to be significantly associated with the PRRS survival trait. Boars with the GG genotype had significantly higher daughters’ survival up to 15 weeks than animals with the AA genotype (68% vs 46%) (Table 4). Remarkably, boars with the GG genotype had daughters with lower *SOX13* expression levels compared to daughters from boars with the AA genotype. Apart from the SNPs described by [25], other SNPs related to the immune system were significantly associated with PRRS survival (Table 4). In SSC16, an intergenic SNP (rs81464083) at 78.72 Mb and located between *IRX2* and *IRX4* genes was also associated with PRRS survival. Boars with the TT genotype had significantly higher daughters’ survival up to 15 weeks than boars TC (89% vs 49%). Another SNP (rs81391061) located at the *ZFHX3* gene was found to be significantly associated to the PRRS survival trait. Boars with the GG genotype had significantly higher daughters’ survival up to 15 weeks than boars with the AA genotype (76% vs 14%) (Table 4).

**Table 4:** **Description of the genetic markers associated with daughters’ survival up to 105 days after the PRRS outbreak depending on sire genotype**

SNP (gene)	Allele (frequency)			Genotype (daughters survival up to 105 days)^4^			Trait
	Resistant	Susceptible	LRT^1^	Homozygous resistant	Heterozygous	Homozygous susceptible	
rs81391061	G (0.85)	A (0.15)	12.71**	GG (0.76)	AG (0.66)	AA (0.14)	Angiogenesis
rs81464083	T (0.68)	C (0.32)	22.62***	TT (0.89)	TC (0.49)	CC (0.43)	Melanoma
rs319560097	T (0.38)	C (0.62)	12.65**	TT (1.00)	CT (0.58)	CC (0.63)	IgG
rs81233340^2^	T (0.90)	G (0.10)	9.84**	TT (0.69)	TG (0.33)		CRP
rs338661853	G (0.87)	A (0.13)	14.64***	GG (0.74)	AG (0.36)		LYM_PHAGO_FITC
rs80904079	A (0.42)	G (0.58)	9.08*	AA (0.71)	AG (0.45)	GG (0.78)	MCV, MCH*
rs80803525^3^	T (0.70)	C (0.30)	6.72*	TT (0.53)	TC (0.79)	CC (0.58)	Lymphocytes
rs342772739	G (0.80)	A (0.20)	3.88*	GG (0.68)		AA (0.46)	γδ T cells
rs323856019	C (0.75)	T (0.25)	5.18*	CC (0.75)	TC (0.55)		Leukocytes
rs343667976	G (0.55)	A (0.45)	2.16 ns	GG (0.59)	AG (0.72)	AA (0.61)	Leukocytes
rs81270251	T (0.70)	C (0.30)	3.64 ns	TT (0.56)	CT (0.72)	CC (0.62)	Neutrophils
rs1107556229 (*CD163*)	A (0.61)	G (0.39)	20.17***	AA (0.54)	AG (0.86)	GG (0.15)	Resistance/susceptibility to PRRSV
rs340943904 (*GBP5*)	T (0.15)	G (0.85)	4.68*	TT (0.87)	GT (0.67)	GG (0.56)	Resistance/susceptibility to PRRSV
MX1_c.-547ins + 250 (*MX1*)	D (0.76)	I (0.24)	4.57*	DD (0.72)	ID (0.44)	II (0.41)	Resistance/susceptibility to PRRSV
rs695254451 (*MMRN1*)	T (0.31)	C (0.69)	3.58 ns	TT (0.74)	TC (0.69)	CC (0.60)	Resistance/susceptibility to PRRSV

^1^*P*-value for the likelihood ratio test of proportional hazard models including or not each SNP: *** = *P* < 0.001, ** = 0.001 < *P* < 0.01, * = 0.01 < *P* < 0.05, = + 0.05 < *P* < 0.10, ns = *P* > 0.10

^2^*CRP* polymorphisms (rs8123340 and rs81285109) were found to be in complete LD with each other

^3^Lymphocytes polymorphisms (rs80803525, rs80899023 and rs80924885) were found to be in complete LD with each other

^4^ Predicted proportion of daughters still alive at 105 days depending on their sire genotype for each SNP: sires having two resistant alleles (Homozygous resistant), sires having both alleles (Heterozygous), and sires having the two susceptible alleles (Homozygous susceptible)

### Cumulative effect of genetic markers on survival to PRRS and slaughter measurements

The eleven markers significantly associated with immunity and survival to PRRS were selected to generate a global immunocompetence index. The MX1_c.−547ins + 250 marker was not included in the index despite being significant in Table 4, because it was not significant when genotyped in the daughters. Table 5 displays the eleven selected SNPs along with the estimated substitution effect for each marker. The global immunocompetence index was calculated for each of the 20 sires multiplying the estimated substitution effect for the number of susceptible alleles for each marker. The best 5 sires had an index value under 1.5, while the worst 5 sires had an index value over 2.5. Hugh differences in daughters’ survival were estimated between best and worst sires: decreased from 94 to 21% as more susceptible alleles were accumulated for the different markers. Sires with less susceptible alleles had daughters with higher IgG levels, lower *SOX13* gene expression, higher carcass weight post-infection with higher backfat thickness, and lower lean meat percentage (Table 6). Heritability estimates for carcass measurements were medium to high, consistent with previous estimations conducted in our Duroc population by [34]. Their results also indicated a genetic relationship between carcass fatness, lean content, and meat pH with a variety of immunity-related traits.

**Table 5:** **Estimated substitution effects for the global immunocompetence index for 11 selected markers**

SNP	Trait	Resistant alelle	Susceptible alelle	Substitution effect
rs81464083	Melanoma	T	C	0.925
rs338661853	LYM_PHAGO_FITC	G	A	0.653
rs319560097	IgG	T	C	0.417
rs81233340	CRP	T	G	0.408
rs1107556229 (*CD163*)	Resistance/susceptibility to PRRSV	A	G	0.405
rs340943904 (*GBP5*)	Resistance/susceptibility to PRRSV	T	G	0.398
rs342772739	γδ T cells	G	A	0.358
rs323856019	Leukocytes	C	T	0.351
rs81391061	Angiogenesis	G	A	0.249
rs80803525	Lymphocytes	T	C	0.218
rs80904079	MCV, MCH	A	G	0.163

**Table 6:** **Regression coefficients for the global immunocompetence index for the different carcass measures at slaughterhouse and heritabilities (standard error)**

Trait	Intercept	Regression coefficient^1^	Heritability (SE)
Carcass weight (kg)	113.55	−3.28^*^	0.63 (0.26)
Carcass lean meat (%)	36.51	2.36^**^	0.61 (0.27)
Backfat thickness 3–4 rib (mm)	38.27	−2.71^***^	0.60 (0.27)
Loin depth 3–4 rib (mm)	40.00	1.66^*^	0.62 (0.25)
Ham fat thickness	25.03	−1.33^*^	0.51 (0.26)
Ham lean meat %	53.00	2.08^**^	0.55 (0.26)
Loin lean meat %	25.91	3.94^**^	0.63 (0.27)
Shoulder lean meat %	49.89	1.92^**^	0.49 (0.27)

^1^*P*-value for the regression coefficient: *** = *P* < 0.001, ** = 0.001 < *P* < 0.01, * = 0.01 < *P* < 0.05, +  = 0.05 < *P* < 0.10, ns = *P* > 0.10

## Discussion

The porcine reproductive and respiratory syndrome virus (PRRSV) is a serious concern for the pig sector as it promotes abortions in sows and respiratory problems, growth retardation, mortality and increases the probability of other diseases in young pigs. To face this threat, breeding animals to produce more robust and disease-resistant pig populations becomes a complementary strategy to the more conventional methods of biosecurity, vaccination, and treatment. Our results showed that the resistance to the PRRSV effects in the affected animals is highly heritable and polygenic. This resistance lies partly in the enhancing role of several genes in the immune response, conferring greater natural resistance to the mortality generated after viral infection.

In our study, SNPs associated with innate and adaptive immunity traits such as C-reactive protein (rs81233340), plasmatic IgG levels (rs319560097), lymphocytes phagocytic capacity (rs338661853), γδ T cells (rs342772739), lymphocytes (rs80803525) and leukocytes (rs323856019) counts in blood, and MCV and MCH (rs80904079) were significantly associated with higher piglet survival to a PRRSV outbreak. Furthermore, previously identified genetic markers (rs1107556229 and rs340943904) in genes (*CD163* and *GBP5*) related to PRRSV entry and immune response have been confirmed to be associated with higher piglet survival after PRSSV infection. Therefore, the inclusion of health-related traits or functionally associated genetic markers in pig breeding programs could contribute to producing more robust and disease resistant animals [25].

A first reaction of the organism to immunological stress such as infections is the acute phase response, an innate, nonspecific systemic reaction triggered by the synthesis and release of pro-inflammatory cytokines, such as interleukin 1 (IL-1), interleukin-6 (IL-6), and tumor necrosis factor-alpha (TNF-α) [35]. Haptoglobin (Hp), C-reactive protein (CRP), serum amyloid A (ASA), and Pig-Major acute phase protein (Pig-MAP) are the main acute phase proteins (APPs) in pigs [36, 37]. Previous studies have determined changes in APPs during experimental or natural PRRSV infection [36–38]. In our study, the SNP (rs81233340) located in the *CRP* gene was significantly associated with daughters’ survival after PRRSV infection. The resistant allele T was associated with higher CRP levels in serum [25] (Additional file 2). Depending on its conformation, CRP functions as a pro-inflammatory molecule by activating the initial phases of the complement system and regulating the release of nitric oxide and synthesis of cytokines. Additionally, CRP acts as an anti-inflammatory agent by regulating the advancement and severity of later stages of inflammation, as well as by modulating apoptosis and phagocytosis processes [39]. Another SNP (rs81391061), located in the *ZFHX3* gene which encodes a transcription factor described as a major regulator of inflammation [40], was also associated with survival after PRRSV infection. In humans, a mutation in this gene has been associated with the expression levels of various inflammation markers such as neutrophil/lymphocyte (N/L) ratio, CRP, and interleukin-6 (IL-6) [40]. In our study, the major allele (G) was associated with higher daughters’ survival and enhanced weight at slaughter.

Pro-inflammatory cytokines play an important role in the pathogenesis of the disease as they are involved in the activation of macrophages. Porcine alveolar macrophages (PAMs) are an important line of defense in front PRRSV infection. These macrophages are the main target cells for PRRSV replication. The *CD163* gene encodes a cellular receptor for PRRSV entry into macrophages [18]. In a previous study the A allele of *CD163*-rs1107556229 was associated with a lower probability of abortion during PRRSV outbreaks [10]. Our results showed that the major allele (A) of rs1107556229 was also associated with higher survival to PRRSV and enhanced weight gain and can be used to select for increased natural resistance to PRRSV. Another marker associated to host resistance against PRRSV infections was the rs340943904 of *GBP5* gene. GBP5 has been described as a marker of interferon gamma induced classically activated macrophages [41]. In pigs, an enhanced induction of antiviral cytokines (IFN-α) and an increased T cell mediated immune response have been described as possible mechanisms for the increased resistance to PRRSV infection in individuals heterozygous for the rs340943904 marker [42]. Our results showed that the minor allele (T) was also associated to higher survival and weights at slaughter following a PRRSV outbreak. Boddicker et al. [8, 9, 43] also demonstrated that the major QTL on SSC4, for which rs340943904 marker is considered a strong candidate causal mutation, had a significant effect on both viremia and weight gain following PRRSV infection in nursery pigs. Therefore, this marker (rs340943904) should be included in the selection program, despite having a low frequency in our population (0.20). Our study also reveals significant interactions between this marker (rs340943904), located within *GBP5,* and another SNP (rs1107556229), located within the *CD163* gene. Dong et al. [16] also detected an interaction between SNPs located within the *GBP5* and *CD163* genes, suggesting a potential biological interaction between both genes.

Porcine IFN-γ production plays also an important role in protection against PRRSV infection. Apart of macrophages, several lymphocyte subsets including γδ T cells have been reported to produce INF- γ during PRRSV infection [44, 45]. γδ T cells can be divided into subpopulations based on their differential expression of workshop cluster 1 (WC1) family members [46]. Additionally, WC1 + cells can be further classified into two main populations, known as WC1.1 + and WC1.2 + , with different ability to respond to specific pathogens and cytokine responses [45]. WC1.1 + and WC1.2 + cells differ in cytokine expression, with WC1.1 + cells preferentially producing IFN- γ, while WC1.2 + cells exhibit higher levels of IL-17. Higher expression levels of *SOX13*, a gene encoding a transcription factor associated with the positive regulation of γδ T cell activation and differentiation [47], has been observed in WC1.2 + cells influencing their differentiation in IL-17 producing γδ T cells [46, 48]. Our results showed that lower levels of *SOX13* expression were associated with higher survival to PRRSV outbreak. Furthermore, the genetic marker rs342772739 was associated with *SOX13* gene expression in our study. This SNP has previously been associated with the percentage of γδ T cells [25]. Boars with the G allele had daughters with lower *SOX13* expression levels compared to daughters from boars with the A allele. This G allele was found to be significantly associated with higher survival to PRRSV and enhanced weights at slaughter. Other markers associated with leukocytes (rs323856019) and lymphocytes (rs80803525) counts in blood were also identified as associated with daughter’s survival to PRRSV in our study. The major alleles in our population (C for rs323856019 and T for rs80803525) have previously been associated with higher leukocytes and lymphocytes counts in blood (Additional file 2), presenting these traits high phenotypic (r > 0.8) and genetic (r > 0.7) correlation coefficients among them and with monocytes and neutrophils counts [25].

Apart from the induction of T cell-mediated immune responses, a humoral response is observed after PRRSV infection in pigs characterized by an initial production of non-neutralizing antibodies followed by the delayed induction of neutralizing antibodies [49]. In our study, higher basal levels of IgG were associated with higher survival to the PRRSV outbreak. Ballester et al. [25] described an SNP (rs319560097) in the proximal region of SSC4 that was associated with the IgG plasma levels (Additional file 2) and it is also associated with higher survival to PRRSV in our study. Indeed, an overlap between this QTL for IgG levels and a previously reported QTL for PRRSV susceptibility [9] was found. Another interesting marker associated with B cells, was the rs338661853. Several studies in mammals have demonstrated that B cells have a significant phagocytic capacity, being able to phagocytose particles including bacteria. The efficient capability of these cells to present antigen from phagocytosed particulate antigens to CD4^+^ T cells, a process more efficient than the presentation of soluble antigens, optimized the induction of humoral response [50]. In our study, the major allele G, previously associated with lower lymphocyte phagocytic capacity [25]  (Additional file 2), was associated with higher survival to PRRSV outbreak. This association may be consistent with literature results showing a delay in and low production of neutralizing antibodies, as well as antibody-mediated enhancement of PRRSV infectivity (reviewed in [49]).

Finally, other markers related with haematological traits, such as MCV and MCH (rs80904079), or melanoma progression (rs81464083) were also associated with survival to PRRSV infection. In our study, the rs81464083 SNP showed the highest association with piglet survival to the PRRSV outbreak. This intergenic SNP is located between IRX genes, which play key roles in the development of both immune and blood cells [51].

Our results indicate that mortality in growing pigs infected by a highly pathogenic PRRSV strain could be reduced through marker selection. Survival upon PRRSV outbreak is heritable and polygenic, and it could be explained by the role of numerous genes in virus entry and the subsequent immune response. These findings enhance our understanding of the genetic control of traits related to immunity and support the possibility of implementing effective selection programs to improve resistance to PRRSV infection and immunocompetence in pigs.

## Supplementary Information

**Additional file 1 Comparison of PRRSV ORF5 sequences. Similarity (%) between the field strain and reference strains.**

**Additional file 2 Boxplots showing the distribution of immunity phenotypes according to associated genetic markers**. (A) Leukocytes counts; (B) IgG levels; (C) MCV; (D) MCH; (E) Lymphocytes counts; (F) CRP; (G) Lymphocytes phagocytic capacity, (H) γδ-T cells.

## Data Availability

The datasets used and/or analysed during the current study are available from the corresponding author upon reasonable request.
