# Supplementary material for: Insights into genetic determinants of piglet survival during a PRRSV outbreak
Source: Vet Res. 2024 Dec 18;55:160. doi: 10.1186/s13567-024-01421-8 (PMC11654192; doi:10.1186/s13567-024-01421-8)
Supplement: Supplementary file 1 — Additional file 1 Comparison of PRRSV ORF5 sequences. Similarity (%) between the field strain and reference strains. [file 13567_2024_1421_MOESM1_ESM.docx]

**Additional file 1. Comparison of PRRSV ORF5 sequences. Similarity (%) between the field strain and reference strains.**

| **PRRSV reference strains** | **Field strain isolated on the studied farm** |
| --- | --- |
| UNISTRAIN PRRS | 83.196 |
| Porcilis PRRS | 81.908 |
| ReproCyc PRRS | 82.237 |
| Suvaxyn PRRS | 82.102 |
| Lelystad strain | 82.072 |
| VR-2332 strain | 63.651 |
| Rosalia strain | 95.222 |
