# Supplementary figures and images for: Insights into genetic determinants of piglet survival during a PRRSV outbreak
Source: Vet Res. 2024 Dec 18;55:160. doi: 10.1186/s13567-024-01421-8 (PMC11654192; doi:10.1186/s13567-024-01421-8)

## Slide 1
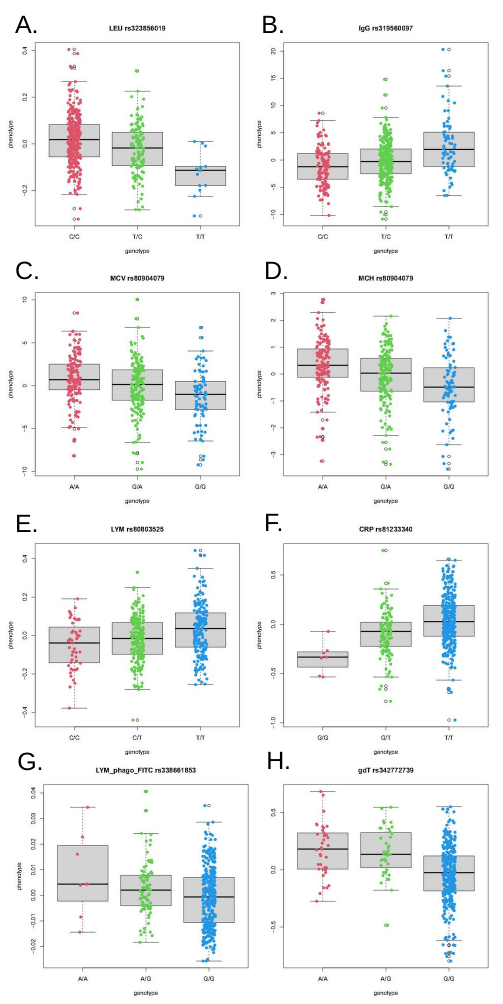

A.
B.
D.
C.
F.
E.
H.
G.

Supplement: Supplementary file 2 — Additional file 2 Boxplots showing the distribution of immunity phenotypes according to associated genetic markers. (A) Leukocytes counts; (B) IgG levels; (C) MCV; (D) MCH; (E) Lymphocytes counts; (F) CRP; (G) Lymphocytes phagocytic capacity, (H) γδ-T cells. [file 13567_2024_1421_MOESM2_ESM.pptx]
